# 

**Published:** 1850-07

**Authors:** 


### CONTENTS

### OF NO. I.

### ORIGINAL COMMUNICATIONS.

### ESSAYS AND CASES.

Art. I.—Singular Case of Monstrosity. By J. W. B. Garrett, M. D., of Macon, Tennessee, - - - 1
Art. II.—A Singular Case of Pregnancy at the Full Period, with Early Death of the Fœtus. By T. C. Osborne, M. D., of Erie, Alabama, - - - - 7
Art. III.—Notes on Miscellaneous Subjects Contained in Foreign and Home Medical Journals. By Theodore S. Bell, M. D., of Louisville, Kentucky, - - 8
Art. IV.—Twins Singularly Connected, &c., &c. By Napoleon B. Anderson, M. D., of Louisville, Kentucky, 19

### REVIEWS.

Art. V.—Medical Jurisprudence. By Alfred S. Taylor, F. R. S., member of the Royal College of Surgeons; and Professor of Medical Jurisprudence and Chemistry in Guy's Hospital. Second American, from the third London edition. With notes and additions by R. Eglesfeld Griffith, M. D.. &c., - - - - 21
Art. VI.—Southern Medical Reports; consisting of General and Special Reports on the Medical Topography, Meterology, and Prevalent Diseases in the following States: Louisiana, Alabama, Mississippi, North Carolina, South Carolina, Georgia, Florida, Arkansas, Tennessee, Texas. To be published annually. Edited by E. D. Fenner, M. D., of New Orleans; member of the American Medical Association, &c., &c., &c., - - - - 26
Art. VII.—A Treatise on the Etiology, Pathology, and Treatment of Congenital Dislocations of the Head of the Femur. Illustrated with Plates. By John Murray Carnochan, M. D. Lecturer on Operative Surgery with Surgical and Pathological Anatomy, - - - 29

ART. VIII.—The American Medical Formulary, based upon the United States and British Pharmacopeias. Including also, numerous standard Formula, derived from American and European authorities. Together with the Medical Properties and uses of Medicines; Poisons, their Antidotes, Tests, etc. Designed for the Medical and Pharmaceutical Student. By JOHN J. REESE, M. D., Lecturer on Materia Medica and Therapeutics in the Philadelphia Medical Institute, &c., - - - - 38
ART. IX.—On the Use and Abuse of Alcoholic Liquors, in Health and Disease. Prize Essay. By WILLIAM B. CARPENTER, M. D., F. R. S., F. G. S., author of "Principles of Human Physiology," etc., etc., - - 39
ART. X.—Essays on the Puerperal Fever and other Diseases peculiar to Women. Selected from the Writings of British Authors previous to the close of the Eighteenth Century. Edited by FLEETWOOD CHURCHILL, M. D., M. R. I. A., Hon. Fellow of the College of Physicians of Ireland; Corresponding Member of the American National Institute, &c., - - - - - - 41
ART. XI.—Transactions of the Medical Society of the State of New York. During its Annual Session, held at Albany, February 5, 1850., - - - - 47

SELECTIONS FROM AMERICAN AND FOREIGN JOURNALS.

Tubercular Exudation into the Lungs (Phthisis Pulmonalis)—Diseased Aortic Valves—Musical Heart Sounds—Ulceration in the Lungs checked by Cod-Liver Oil, - 73
Pneumonia in Children, - - - - - - 76
Aneurism of the Popliteal Space treated successfully by Compression, - - - - - - 79
Method of Depriving Quinine of its Bitterness, - - 82
On Gleet and its treatment, - - - - - - 83
On the Emission of Urine, as observed in an Individual suffering from Ectopia of the Bladder, - - - - 83
On a Case of the Spontaneous Reproduction of Cowpock, 84

ORIGINAL INTELLIGENCE.

The American Medical Association, - - - - 85
Premium for an Essay on Physiology, or Medical Chemistry, - 86
Prize Essay on Nostrums, - - - - - 87
A New Medical School, - - - - - - 89

